# The hidden costs: Identification of indirect costs associated with acute gastrointestinal illness in an Inuit community

**DOI:** 10.1371/journal.pone.0196990

**Published:** 2018-05-16

**Authors:** Nia King, Rachael Vriezen, Victoria L. Edge, James Ford, Michele Wood, IHACC Research Team, Rigolet Inuit Community Government, Sherilee Harper

**Affiliations:** 1 Department of Population Medicine, University of Guelph, Guelph, Ontario, Canada; 2 Department of Food, Agriculture, and Resource Economics, University of Guelph, Ontario, Canada; 3 Indigenous Health Adaptation to Climate Change Research Team, Guelph, Ontario, Canada; 4 Priestley International Centre for Climate, University of Leeds, Leeds, United Kingdom; 5 Department of Health and Social Development, Nunatsiavut Government, Goose Bay, Labrador, Canada; 6 Rigolet Inuit Community Government, Rigolet, Labrador, Canada; University of Saskatchewan, CANADA

## Abstract

**Background:**

Acute gastrointestinal illness (AGI) incidence and per-capita healthcare expenditures are higher in some Inuit communities as compared to elsewhere in Canada. Consequently, there is a demand for strategies that will reduce the individual-level costs of AGI; this will require a comprehensive understanding of the economic costs of AGI. However, given Inuit communities’ unique cultural, economic, and geographic contexts, there is a knowledge gap regarding the context-specific indirect costs of AGI borne by Inuit community members. This study aimed to identify the major indirect costs of AGI, and explore factors associated with these indirect costs, in the Inuit community of Rigolet, Canada, in order to develop a case-based context-specific study framework that can be used to evaluate these costs.

**Methods:**

A mixed methods study design and community-based methods were used. Qualitative in-depth, group, and case interviews were analyzed using thematic analysis to identify and describe indirect costs of AGI specific to Rigolet. Data from two quantitative cross-sectional retrospective surveys were analyzed using univariable regression models to examine potential associations between predictor variables and the indirect costs.

**Results/Significance:**

The most notable indirect costs of AGI that should be incorporated into cost-of-illness evaluations were the tangible costs related to missing paid employment and subsistence activities, as well as the intangible costs associated with missing community and cultural events. Seasonal cost variations should also be considered. This study was intended to inform cost-of-illness studies conducted in Rigolet and other similar research settings. These results contribute to a better understanding of the economic impacts of AGI on Rigolet residents, which could be used to help identify priority areas and resource allocation for public health policies and programs.

## Introduction

Acute gastrointestinal illness (AGI), defined as diarrhea or vomiting not due to a chronic condition, pregnancy, or alcohol/drug use [1], is a leading contributor to the disease burden [2,3] and economic costs of illness in many countries [4–6]. Multiple studies have therefore investigated the incidence and burden of AGI in developed countries to inform public health decision-making with respect to improving health outcomes, increasing quality of life, and reducing healthcare costs for both individuals and governments [1,3,7,8]. These studies report that although the clinical course of AGI in developed countries is characteristically mild, the large incidence of disease results in substantial morbidity-associated costs; the estimated per-capita annual cost of gastroenteritis converted to 2016 USD was up to USD$174 in the United States in 2013 [9,10] and USD$115 in Canada in 2001 [11]. Given these substantial costs, cost-of-illness studies are emerging as crucial tools in understanding the impact of illnesses on society in order to inform public health policy development, prioritization, and decision-making (S1 Table) [12].

Cost-of-illness estimates are typically calculated based on the estimated annual number of cases and a unit cost per case [5,6,12]. This unit cost commonly includes direct medical costs, which are derived on the basis of healthcare usage and include the costs of resources consumed in providing care, and indirect lost productivity costs, which are calculated using the average number of missed days of employment and the average wage rate [11–13]. Direct non-medical costs, which include costs associated with transportation to the medical facility and household expenditures related to the illness, are occasionally incorporated into the unit cost [2,4,12,14]. Although these methods provide a broad estimate of the economic costs of illnesses, they overlook many indirect costs that vary substantially between populations due to socio-demographic, cultural, geographic, and lifestyle differences [5,11]. While some studies are beginning to incorporate quality of life measures into comprehensive cost assessments, the majority of cost-of-illness studies that include the indirect costs of AGI account only for missed paid employment and not for other indirect costs such as those associated with reduced quality of life, time away from other unpaid duties including housework and childcare, and reduced food security [12,15]. These under-researched indirect costs have substantial effects on individual quality of life and can further exacerbate physical health outcomes [16]. Consequently, the current approaches used to evaluate costs of illness often do not capture the broader impacts of AGI on individuals [11,15]. To address this limitation, researchers have suggested that case-based studies should be employed to provide detailed information on the costs of illnesses, both direct and indirect, in specific settings based on micro-level economic situations [5,11,15,17].

Researchers have noted that case-based studies are particularly important when working with populations that operate within a setting-specific economy that varies substantially from the general economy [5,11,15,17]. For instance, the mixed subsistence-based economy characteristic of many rural Indigenous populations, in which households rely on a combination of wage labour and the subsistence outputs of hunting, fishing, trapping, and gathering, combined with the geographic and cultural differences from non-Indigenous communities, creates a need for case-based studies to gain an accurate understanding of the costs of illness within these communities [14,18–20].

Inuit, one of the three constitutionally recognized Aboriginal groups in Canada [21], typically operate within a mixed subsistence-based economy; missing both paid employment and subsistence activities due to illness can therefore have negative individual- and household-level economic impacts [20]. Furthermore, some Inuit populations have a higher burden of disease compared to other populations, in part due to the persisting disparities in the social determinants of health such as income, education, and food security, which are interwoven with the legacy and ongoing experience of colonization and discrimination [18,19,22,23]. Low healthcare accessibility and availability in remote Inuit communities further exacerbate existing health disparities [18,19,24]. Furthermore, due to their intimate relationships with the natural environment and their reliance on, and love for land-based subsistence activities, Inuit populations bear an unequal burden of the physical and mental climate-health impacts [25,26], which include disproportionate rates of AGI [1]. A recent study reported that the annual incidence of AGI in two Inuit communities varied from 2.9 to 3.9 cases per person-year [1], whereas studies conducted elsewhere in Canada reported rates between 1.2 and 1.3 cases per person-year [3,8,11]. Lastly, the limited local healthcare provision, geographic remoteness, small population sizes, and expensive travel costs to access medical treatments typical of most Inuit communities render the healthcare costs in these communities some of the most expensive in world, which could contribute to a further increased AGI cost burden [14,27,28].

These differences in economic systems, social determinants of health, AGI incidence, and healthcare costs in Inuit communities as compared to other Canadian communities could substantially influence the costs of illnesses, including direct monetary costs and impacts on quality of life. However, many of the context-specific costs of AGI remain unaccounted for given that most cost-of-illness studies conducted in Inuit communities are based on models developed for non-Inuit communities [20]. These unaccounted variations are especially relevant for public health officials who rely on these estimates when making evidence-based decisions [11,20,24]. Thus, community-level case studies that gather context-specific social and economic information in Inuit communities are needed to provide a comprehensive understanding of the costs of illness particular to these settings [14,20]. Reflecting these research needs, this exploratory study aimed to identify and characterize community-level indirect costs of AGI, and explore factors associated with these indirect costs, that should be included in comprehensive cost-of-illness studies in the Inuit community of Rigolet, Canada. A mixed-methods study design was used, and consisted of qualitative research to identify and characterize indirect costs of AGI, and quantitative research to explore how often these indirect costs were incurred at a population-level. The study intended to inform the development of a case-based context-specific study framework that is meaningful to both Indigenous and non-Indigenous policy-makers, and could be used to evaluate indirect costs of AGI in Rigolet and adapted for other Inuit communities and self-limiting illnesses.

## Methods

### Study location

Inuit live primarily within four settled Inuit land claim regions in northern Canada: Inuvialuit Settlement Region, Nunavut, Nunavik, and Nunatsiavut. The Labrador Inuit Land Claim Settlement Area, led by the Nunatsiavut Government (NG), is situated on the northeast coast of Labrador and has a population of 2617 residents living among five communities: Rigolet, Nain, Makkovik, Postville, and Hopedale [29]. This study took place in the community of Rigolet, which has a growing population of approximately 305 residents, 92% of whom identify as Inuit [29]. Rigolet is a remote community and remains without external road access. Transportation is therefore dependent on a commercial year-round plane service, a seasonal ferry service, personal boating in the summer, and snowmobiling in the winter. A single retail store stocks a variety of fresh and processed foods brought in by air or by water in the summer.

Rigolet Inuit have a deep connection to the natural environment and value their traditional lifestyle that relies on the land and water surrounding the community (locally referred to as “the land”) [25]. Spending time on the land while hunting, trapping, fishing, and gathering foodstuffs is an integral part of the population’s livelihoods, lifestyle, well-being, and cultural identity [25,27,30]. These activities have also been linked to reduced stress, increased productivity, and improved physical and mental well-being [25,30–32]. To participate in multi-day trips on the land many residents have cabins on the land that can be accessed by boat in the summer or snowmobile in the winter.

The provincial government, through the Labrador-Grenfell Health Authority, provides Rigolet residents with primary healthcare, pharmaceutical medicines, and clinical mental health services. Rigolet has a primary healthcare clinic staffed by two resident nurses and a visiting physician approximately every six weeks. Residents requiring emergency care or specialized services must travel to a southern health centre, typically in Goose Bay, Labrador (1/2 hour flight) or St. John’s, Newfoundland (two flights; 1/2 hour and 1.5 hour). A previous study conducted in Rigolet reported that 4.8% of survey respondents self-reporting AGI sought medical care from a clinic or hospital; none of these participants required treatment from a southern health centre [24]. Traditional Indigenous healthcare is not commonly used for AGI in Rigolet; a previous study found that only 2.5% of surveyed Rigolet residents would use traditional Inuit medicine (e.g. teas, herbs) to treat AGI, and that none would consult a traditional Inuit healer [30]. Medical services are financed by the Province of Newfoundland and Labrador’s Medical Care Plan, as well as the Non-Insured Health Benefits (NIHB) Plan for Inuit beneficiaries administered by NG. The NIHB Plan covers a range of prescription and over-the-counter medications as well as the direct medical and non-medical costs incurred by individuals when travelling to a southern health centre for diagnosis or treatment. The NIHB Plan does not cover any indirect costs of illness, such as extra food expenses, childcare costs, or lost wages.

### Community engagement

This study emerged from an ongoing partnership between university researchers and the community of Rigolet, the Rigolet Inuit Community Government, and the NG. In 2006, the community of Rigolet and Nunatsiavut health representatives identified water safety and waterborne disease, including AGI, as regional climate-sensitive health priorities [27,33]. Since then, our team of university and community researchers, and government and NGO partners have worked together to investigate several community-identified dimensions of AGI, including the burden of disease, healthcare use, and climatic variables associated with AGI occurrence [1,24,30,33].

Considering the unethical practices that have characterized some past research in Inuit communities, our interdisciplinary team uses community-based and community-led participatory research approaches, in which decision-making and ownership are shared with the community, bi-directional knowledge sharing is promoted, and research findings are co-created and disseminated in a relevant and beneficial manner [27,34–36]. Accordingly, this study was co-designed by an interdisciplinary team of Inuit and non-Inuit researchers, government representatives, and community leaders. The integration of community perspectives and Inuit knowledge was considered essential to the research process, and therefore ongoing active and meaningful community involvement was emphasized at all stages of the research process.

The Tri-Council Policy Statement on the ethical conduct of research involving the First Nations, Inuit, and Métis, was used as the ethics framework for this research [37]. In addition, the principles of respect, reciprocity, relevance, and responsibility, which are central to conducting research with Indigenous peoples [38], were also prioritized in this project, which helped to establish positive, stable, and trusting relationships among the team [27,34–36]. Written informed consent was obtained from all interviewees and survey participants. Both quantitative and qualitative protocols were approved by the NG Research Advisory Committee, a committee of NG representatives that consults communities throughout the research approval process, and the Research Ethics Boards at the University of Guelph and McGill University. The Health Canada Research Ethics Board approved the use of the burden of illness surveys. The study was also formally approved by the Rigolet Inuit Community Government.

### Qualitative Data Collection & Analysis

The qualitative portion of this study drew from fifteen qualitative in-depth interviews, two group interviews, and three key informant interviews conducted in Rigolet and Goose Bay in July 2015, and nine AGI-case interviews conducted in Rigolet in November 2012 (Table 1). All qualitative data were collected in-person alongside an Inuit research associate at a location agreed upon by the interviewees. All interviews were conducted in English according to participant preference. Interview guides were formally pre-tested by University of Guelph academics and NG partners, and were adapted where needed. The Inuit community research associate also piloted the interview guide in Rigolet with two community members. Locally-relevant terms for AGI, including ‘bad stomach,’ ‘stomach bug,’ and ‘stomach illness,’ were used throughout the interviews. Prompts were used if necessary to obtain additional information.

**Table 1:** Demographics of community in-depth, group, and AGI-case interviewees.

**Gender**	n	%
Female	21	65.6%
Male	11	34.4%
**Age**		
0–20*	2	6.2%
20–39	9	28.1%
40–59	15	46.9%
60+	6	18.8%

*Interviews with minors were conducted with their guardian as their proxy respondent; minors were present throughout the interview.

#### Community & group interviews

Interviewees included residents from diverse backgrounds and positions within the Rigolet community. The community research associate used maximum variation sampling, which involves purposefully choosing a wide range of participants to obtain a variation on dimensions of interest, in order to maximize the diversity of perspectives and to obtain a comprehensive understanding of the topic [39]; however, participation was open to any interested individual in Rigolet. A combination of group and individual interviews was used to capitalize on the strengths of both approaches. Group interviews account for the Indigenous way of knowing, which is linked to process of individuals collectively constructing their understandings by experiencing their social being in relation to others [40]. Additionally, intragroup interactions inherent in group interviews can stimulate participants to consider and reflect upon forgotten or unconsidered details that would otherwise be overlooked [41]. However, individual perspectives may be silenced in group settings if participants choose to conform to the popular opinion rather than state their own perspectives [42,43]. In order to minimize this potential, the community research assistant carefully considered personalities and social factors, such as age, social status, and education level, when creating the groups to ensure that all participants would feel free to express their opinions and perspectives [42–44]. Any prospective participant identified by the local Inuit research associate based on past research experience as particularly outspoken was interviewed individually. Consequently, by creating open and comfortable group interview environments, we were able to gain novel group insights which were subsequently complemented with individual perspectives from individual interviews [41,45]. These interviews were collaboratively conducted by the first author and Inuit research associate. Semi-structured interview guides [46] included open-ended questions capturing data on community health-seeking behaviours and the impacts of AGI on productivity, daily activities, mental well-being, and social welfare in order to gain an understanding of all indirect costs of AGI incurred by residents (S1 File). Individual interviews (n = 15) lasted a total of 311 minutes and group interviews (n = 8 interviewees) lasted a total of 50 minutes.

#### AGI-case interviews

The study also used data from nine AGI-case interviews that were conducted alongside the quantitative survey in Rigolet in November 2012 as part of another study [30]; AGI-case interviewees were purposively selected from those self-reporting AGI within the September 2012 quantitative survey (see Quantitative Data Collection & Analysis below) in order to reflect the case attributes. These interviews were conducted by the last author and captured information on the lived experience of AGI in Rigolet. None of these AGI-case participants were re-interviewed in July 2015. AGI-case interviews (n = 9) lasted a total of 325 minutes.

#### Key informant interviews

Key informants were purposively selected based on their job description, invited via email for an interview, and interviewed in-person at each interviewee’s office. Key informant interviews (n = 3) with NG employees in Goose Bay were conducted in July 2015 and lasted a total of 90 minutes. Key informants were asked about a variety of community and governmental costs of AGI (S1 File).

#### Qualitative analysis

All interviews were audio recorded with consent, transcribed by the first author, and manually reviewed for accuracy. A five-step comparative thematic analysis approach was employed [47,48]. Steps included data familiarization, code identification, theme identification by collating codes using concept maps and reflective memos, codebook development and transcript coding, and code review to test the reliability of the analysis [47–49]. Qualitative data analysis software (Atlas.ti, version 6) helped with data organization and quotation retrieval, and to provide an analysis audit trail [48]. According to the principles of community-based participatory research, the first author worked with the local Inuit research associate to collaboratively identify relevant codes and themes. Additionally, member checking was conducted, during which a list of preliminary findings and quotations were provided to study participants to solicit feedback, and to ensure the accuracy, authenticity, and relevancy of results to the research context.

### Quantitative Data Collection & Analysis

Quantitative analyses were used to explore associations between predictor variables and indirect costs to help contextualize the qualitative results at the population-level. Quantitative data were collected from two retrospective cross-sectional burden of illness surveys conducted in Rigolet in September 2012 and May 2013 as part of a larger burden of AGI study [1]. The survey design, sampling procedure, administration, and survey questionnaires are detailed elsewhere [50]. The small population size of Rigolet rendered a census sample reasonable. In September 2012, results from 226 of the 245 individuals within the community during the study period were obtained, compared to 236 of the 249 individuals present in May 2013. The AGI case definition used was vomiting and/or diarrhea in the past 14 days, not due to pregnancy, medication/alcohol/drug use, or diagnosed chronic conditions [1,51]. There were 30 self-reported cases of AGI in September 2012 and 32 self-reported cases in May 2013 [1].

Quantitative survey data were analyzed using Stata/IC 14.1 for Mac (StataCorp., USA) using a significance level of α = 0.05. Data from participants responding ‘unsure’ or ‘refused to answer’ were excluded from that question’s analysis (S2 Table). To identify factors potentially associated with indirect costs of AGI, univariable unconditional logistic regression examined potential associations between exposure and outcome variables. Following a mixed methods approach [52], preliminary qualitative findings informed the development of the univariable models: primary indirect costs and cost variables identified in qualitative interviews were used in the models. Indirect costs captured by the survey and considered in the models included missed subsistence activities (captured only for participants reporting AGI within the previous two weeks), recent visits to the land or cabin (captured for all participants), money spent on country food, money spent on store food, number of meals including country food, number of meals including store food, overall life satisfaction, and sense of community belongingness. Season and AGI status were also included in the models. Table 2 outlines the outcome and exposure variables included in the quantitative analysis, as well as the justification for including each variable. Univariable unconditional logistic regressions were used to analyze the effect of each predictor variable on each indirect cost outcome. A random intercept was used to control for household-level clustering.

**Table 2:** Quantitative predictor and outcome variables used to analyze the indirect costs of acute gastrointestinal illness (AGI) in Rigolet, Nunatsiavut, Canada.

Cost category	Indirect cost (description)	Predictor: Justification	Type
Missed subsistence activities	Missed subsistence activity* (Whether the individual missed a subsistence activity due to AGI in the previous 14 days)	**Season:** Interviewees stated that the season may influence the likelihood of them suffering through their symptoms to go out on the land.	Categorical/ dichotomous
	Recent visit to the land or cabin (Whether the individual went out on the land or visited a cabin in the last month)	**Season:** Participants may be more likely to visit the land or their cabin during certain seasons given that country and retail food availability vary seasonally [57]. **AGI status:** Interviewees stated that AGI would prevent some individuals from participating in subsistence activities.	Categorical/ dichotomous
Altered diet	Money spent on country food; Money spent on retail food (Expenditures on country food or retail food over the last week) Number of meals including country food; Number of meals including retail food (Frequency of consumption of country food or retail food over the last month)	**Season:** Country and retail food availability vary seasonally, meaning that participants may be more reliant on certain food sources in different seasons [57]. **AGI status:** Interviewees stated that individuals sick with AGI may rely more heavily on retail foods that are easier to digest; this is corroborated by another study [30]. **Recent visit to the land or cabin:** Interviewees stated that not being able to spend time on the land would result in decreased country food availability and therefore increased reliance on store food.	Categorical/ ordinal
Mental well-being costs	Overall life satisfaction (Overall rating of life satisfaction)	**Season:** Rigolet residents’ mental well-being is more vulnerable in certain seasons [73]. **AGI status:** Interviewees stated that AGI had a negative impact on mental well-being. **Recent visit to the land or cabin:** Interviewees stated that spending time on the land was crucial to maintaining mental well-being; other studies report that decreased subsistence activity productivity is linked with decreased mental well-being [22,24].	Categorical/ dichotomous
Social welfare costs	Sense of belonging within the community (Overall rating of feelings of belongingness within the community)	**Season:** Rigolet residents’ mental well-being is more vulnerable in certain seasons [73]; feelings of community belongingness likely contribute to residents’ mental well-being. **AGI status:** Interviewees stated that AGI decreased individuals’ ability to participate in social activities, which may impact their sense of community belongingness.	Categorical/ dichotomous

*Only asked to participants who reported AGI in the prior 14 days

## Results

**Fig 1:**
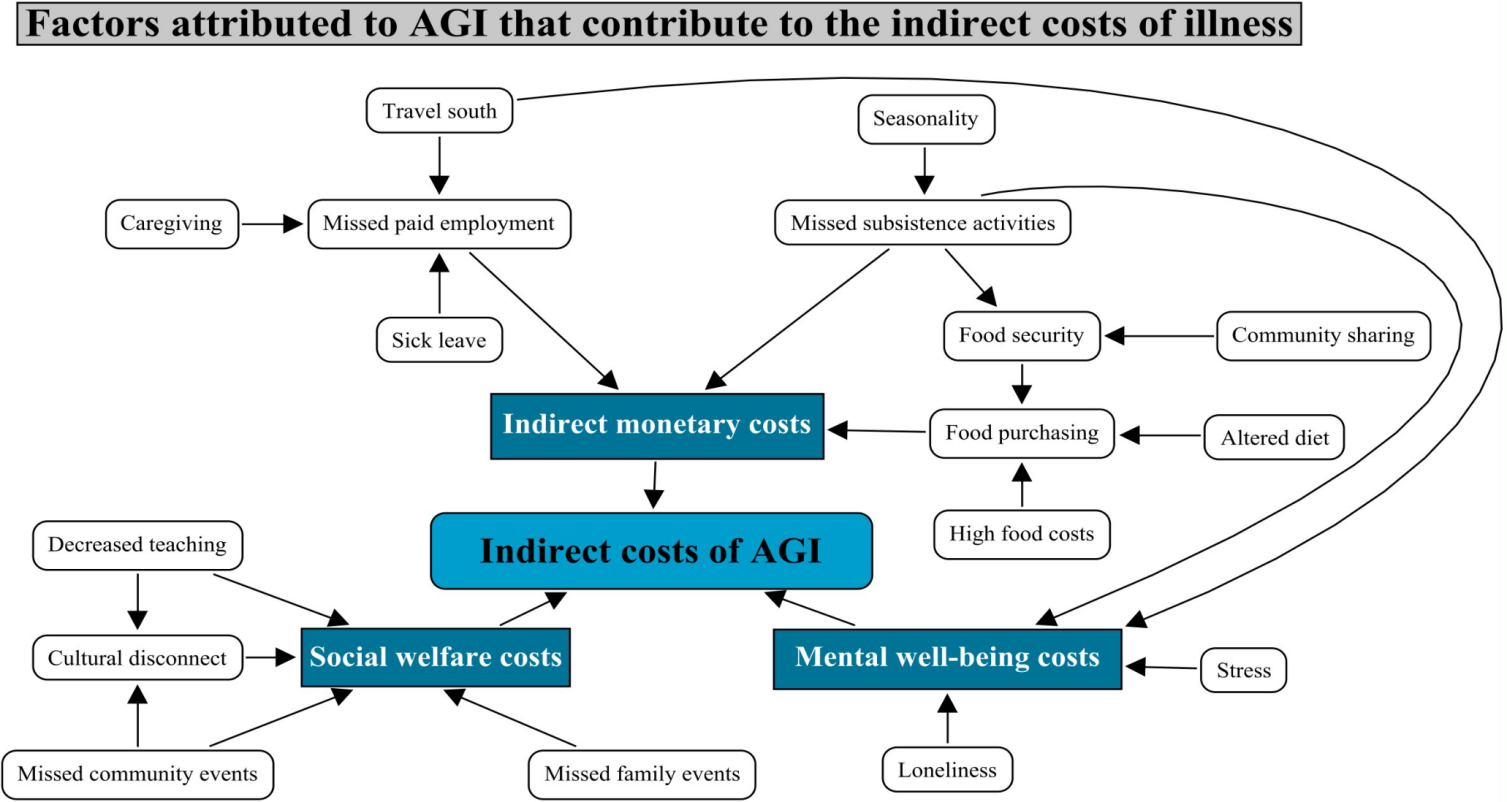
An overview of perceived contributors to the indirect costs of AGI as reported by interviewees in Rigolet and Goose Bay, Labrador, Canada.

As illustrated in Fig 1, the primary indirect cost contributors of AGI identified by interviewees were indirect monetary costs attributed to missed paid employment, missed subsistence activities, and an altered diet; mental well-being costs; and social welfare costs (Fig 1; S3 Table). As shown in the figure, these cost contributors were inter-related, meaning that one cost contributor often exacerbated others.

### Indirect monetary costs: “The economic issue is big”

#### Missed paid employment: “They’ll have to go without pay”

Many interviewees described missing work due to AGI, often resulting in economic consequences attributable to lost wages: *“Well*, *they [those with AGI] would definitely be missing work*. *Like*, *I wouldn’t want to go to work sick like that*.*”* As one young mother stated, *“I have no doubt that a lot of people miss a lot of work because of [AGI]*.*”* Interviewees described that economic losses may also accrue if the working individual must stay home to care for a sick loved-one: *“I mean especially with younger kids*, *you would have to take care of them*, *you can’t leave them and go on*, *so you would be stuck too*.*”* As a whole, interviewees reported that costs associated with missing paid employment due to AGI had *“a huge impact on families*.*”* This impact reportedly varied in magnitude according to the individual’s job, given that in contrast to most government positions, many short-term and contract jobs in Rigolet did not provide sick leave coverage. Due to the potentially substantial economic impacts associated with missing work, some interviewees reported working through AGI symptoms to avoid missing paid employment: *“I don’t have time to be sick anyways*, *I’ve been sick a few times in the winter but you don’t stop working or nothing*.*”*

#### Missed subsistence activities: “The store is there but selection is limited, and so [subsistence] activities are very important”

In addition to missing paid employment, most interviewees and survey participants cited missing subsistence activities as a major economic cost associated with AGI: 10.1% [4.8%-20.1%] of survey participants who had been sick with AGI reported missing a planned subsistence trip due to the symptoms. Additionally, survey participants who had been sick with AGI were significantly less likely (OR = 0.46, p = 0.044) than non-sick participants to have recently visited the land or cabin (Table 3). Interviewees described the indispensable role of subsistence activities in food and economic security, as well as physical and mental well-being, and overall quality of life. Consequently, many interviewees reported attempting to *“suffer”* through the symptoms in order to minimize the number of missed subsistence trips: *“Most of the time people at least try to go out [even if they are ill]*.*”*

**Table 3:** Results from the univariable logistic regression models (for those variables with p<0.10) based on survey data from Rigolet, Nunatsiavut, Canada.

Predictor	Outcome (Indirect Cost)	Odds ratio (p-value)	95% Confidence Interval
AGI case status (ref. = No AGI)	Number of meals containing retail meat	2.89 (0.012)	(1.26, 6.64)
	Recent visit to the land or cabin	0.46 (0.044)	(0.22, 0.98)
Recent visit to the land or cabin (ref. = No visit)	Number of meals containing retail meat	0.46 (0.010)	(0.25, 0.83)
	Number of meals containing country meat	1.91 (0.023)	(1.10, 3.32)
Season (ref. = September)	Number of meals containing country meat	19.55 (<0.001)	(9.07, 42.10)
	Number of meals containing retail meat	10.09 (<0.001)	(5.04, 20.22)
	Recent visit to the land or cabin	0.41 (<0.001)	(0.25, 0.67)
	Overall life satisfaction	1.64 (0.018)	(1.09, 2.47)

Missing subsistence trips due to illness also reportedly resulted in decreased food availability for the individual and their household in both the short- and long-term, as it *“could impact what you have in your freezer for later on*.*”* One key informant explained:

When it comes to fishing for salmon and char and things like that, that impacts not only that period of time but throughout the winter as well because that’s the winter stores of food for a lot of families, it is how they get their proteins, and berries, and things like that that actually sustain them through the winter months.

Survey participants who had recently visited the land or cabin consumed country meat significantly more often (OR = 1.91, p = 0.023), and retail meat significantly less often (OR = 0.46, p = 0.010), than those who had not recently visited the land or cabin (Table 3). Additionally, without provisions obtained from subsistence activities, interviewees reported that they would be forced to purchase expensive retail foods, which could have household-level economic consequences:

It’s something that not everybody [in Canada] would think about because usually you can go to the store and get everything you need and it’s not a big deal [in southern Canada], but that’s not necessarily the case for our small communities where people are very reliant on hunting.

Interviewees reported that the retail store had limited selection and was extremely expensive. The food obtained from a single hunting trip could *“feed [a] family for months*,*”* but one interviewee described that *“money only stretches so far”* when purchasing retail foods. One hunter quantified this impact: *“you go kill a goose*, *you don’t spend the $50 to get a turkey anymore*. *You got the goose already*, *so that’s $50 you save*.*”* Nevertheless, retail food expenses were not significantly lower for survey participants who had recently visited the land or cabin as compared to those who had not (p = 0.694).

Lastly, interviewees described that the impacts of missing subsistence activities varied seasonally. As one hunter reported:

[The impact of AGI] Depends on the season, if it’s in the spring and it’s goose hunting time, you only got a short period of time, you’ve got a month. And then if you miss the peak time that the birds come through, you’ve lost that part […] So there are big consequences in certain times of the year. Wintertime, not so bad, you have a longer period of time to get your wood and gather food and partridges […] but in the summertime and springtime, you don’t have that time.

Interviewees therefore reported that individuals would be less likely to miss subsistence activities in the spring; however, the survey results showed no significant difference in missing subsistence activities due to AGI symptoms between seasons (p = 0.701). Nevertheless, certain seasonal associations were significant within the survey results: visits to the land or cabin (OR = 0.41, p<0.001) were less common in May compared to September (Table 3). Conversely, consumption of both country meat harvested from subsistence activities (OR = 19.54, p<0.001) and retail meat purchased from the store (OR = 10.09, p<0.001) were significantly higher in May compared to September (Table 3).

#### Altered diet: “They would probably just eat bananas, applesauce, whatever, something that’s not too heavy”

Almost all interviewees described altering their diet when sick with AGI to reduce symptom severity. Interviewees reported an increased reliance on retail foods, such as bananas and applesauce, as they were deemed to be easier to digest than country food: “*If [you’re] sick with the stomach flu you might not be able to tolerate so much of the meats and fishes*. *You might have to be buying things […] So yes*, *it could be more expensive*.*”* Survey results indicated that AGI case status did not significantly alter the number of meals containing country meat (p = 0.584); however, AGI cases were significantly more likely to consume meals with store meat (OR = 2.89, p = 0.012) (Table 3). Lastly, as one participant stated, many individuals also described that: *“Well I think when most people have the stomach bug*, *they don’t eat at all*. *That’s my experience anyways*.*”* Consequently, one interviewee stated: *“If anything*, *you’re saving money*.*”*

### Mental well-being costs: “There is a certain helplessness that sort of goes with that [AGI]”

Interviewees reported that AGI impacted individuals’ quality of life through decreased mental well-being: one interviewee described a *“stress factor that comes into play”* when sick, as *“people begin to worry”* about their health and missing paid employment. Another female interviewee described that even after recovery *“you usually [physically] get back to normal*, *[but] you usually don’t forget [about the stress]*.*”* Many interviewees also reported loneliness associated with being sick, as *“nobody wants to be around you*, *[AGI] spreads so quickly here*.*”* However, ratings of overall life satisfaction did not significantly differ between participants who reported AGI and those who did not (p = 0.48).

Interviewees reported that spending time on the land *“revived [their] spirit”* and *“restored a sense of balance*.*”* Subsistence activities were viewed as a crucial component to mental, emotional, and spiritual well-being; limitations on an individual’s ability to spend time on the land were therefore the most commonly identified mental well-being impact of AGI and resulted in feelings of sadness and frustration. Moreover, interviewees described the importance of subsistence activities in defining an individual’s self-worth. One key informant explained:

[AGI] can be limiting because you can’t hunt, you aren’t able to provide for your family. And there is a bit of a feeling of helplessness because people take a great amount of pride in being able to provide for their families, and if you can’t do that because you’re ill, there is a certain helplessness that goes with that.

A decrease in country food consumption was also reported to negatively impact individuals’ mental and spiritual well-being. One community health official explained:

You can’t even compare a duck to a chicken, or a goose to a turkey. Because when you eat a chicken or a turkey, that is something that you buy from the store, but when you go and gather it yourself and you have that whole experience, and that connection, and you come back, it’s like you’re feeding your soul, not just your body.

Lastly, overall life satisfaction varied seasonally, and was significantly higher in May as compared to September (OR = 1.64, p = 0.018).

### Social welfare costs: “It really does impact peoples’ place in the community”

Interviewees identified many social welfare costs associated with missed family and community activities due to AGI. As one key informant described, when sick with AGI *“you can’t actually partake in some of the things you’d like to partake in because you’re so uncertain as to if you are going to become symptomatic during an event*,*”* meaning that *“you have no social life*.*”* Interviewees highly valued social activities and therefore missing such activities was considered a social welfare loss resulting in decreased quality of life. However, the survey results did not demonstrate a significant association between AGI and overall sense of community belongingness (p = 0.85).

### Case-based indirect cost-of-illness study framework

**Fig 2:**
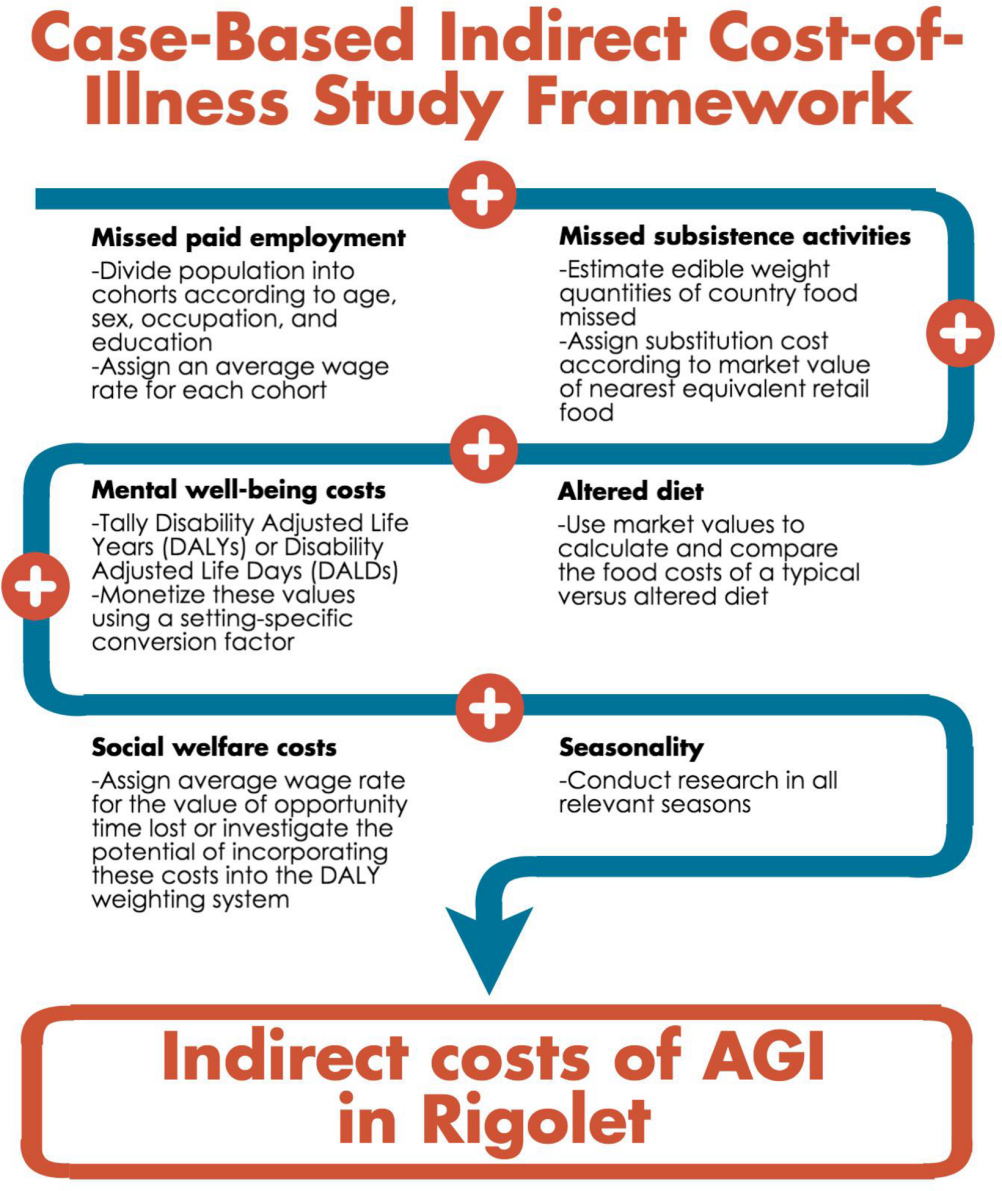
Suggested case-based indirect cost-of-illness study framework that accounts for all indirect costs of illness reported by interviewees in Rigolet and Goose Bay, Labrador, Canada.

The primary indirect cost contributors discussed by participants were incorporated into a case-based context-specific study framework in Fig 2. By accounting for each of these components, this framework can be used to more accurately evaluate the indirect costs of AGI in Rigolet.

## Discussion

This study provides insights into unique factors that contribute to the indirect costs of AGI borne by Rigolet residents. The developed cost-of-illness study framework incorporates both Inuit community perspectives, as well as Western science-based cost-of-illness literature, thus ensuring that the framework is meaningful for both Indigenous and non-Indigenous decision-makers. This framework can be used to generate more accurate estimates of the context-specific costs of AGI in Rigolet.

Interviewees’ reports of lost productivity due to AGI were reflected in recent research conducted in Rigolet, which found that AGI caused sick individuals to miss an average of 0.21 days of work in a two-week period [24]. Other studies equally corroborate interviewees’ reports that lost wages may also result from a caregiver needing to miss work [5,6,11,24,53]. As such, both interviewees and previous studies indicated the need to account for the lost productivity associated with AGI [15,54]. One common method used to quantify these productivity losses is the human capital approach wherein missed paid employment time is valued at the market value of that individual’s future contribution to production if the individual had continued to work in full health: each day of missed paid employment is valued at the average wage lost for an individual in that particular setting [5,6,11,13,55]. However, interviewees described that the magnitude of the economic consequences associated with missed paid employment differed according to individual characteristics, in that some might benefit from sick-leave coverage. Such a consideration is important when investigating individual-level costs of illness; however, in community- or society-level studies, a cost in the form of lost wages or lost production is incurred if an employee becomes ill regardless of their sick-leave coverage status, meaning that such a consideration becomes less important. Nevertheless, other authors similarly report that in using average wage rates to value lost productivity, studies overlook factors that influence both wage rates and disease incidence, such as age, sex, occupation, and education [2,56]. The Dutch cost research guidelines suggest that such factors should be taken into account by grouping individuals into cohorts and subsequently assigning cohort-specific average wage rates [2,56,57]. In conducting case-based studies, this strategy could be employed to more accurately value productivity losses [56].

As substantiated by other studies, interviewees highly valued subsistence activities for their contributions to food and economic security, and quality of life through physical and mental well-being [20,25,27,30,58]. One northern Canadian study reported that through subsistence activities, households can convert each cash dollar into $3.00 of consumer benefit [58], which could explain why many interviewees described attempting to *“suffer”* through the symptoms.

Interviewees also highlighted the decreased food availability that may result due to missed subsistence activities. Inuit populations experience high levels of food insecurity compared to other Canadian locales; community studies have reported a prevalence of food insecurity between 50% and 80% in the Canadian North [59–62]. Country foods hold substantial cultural, nutritional, and social value in these communities, and their consumption is associated with decreased incidence of many diseases including Type 2 diabetes and cardiovascular diseases [61–64]. As compared to country foods, nutritious retail foods are higher in price and are often spoiled due to the long transit time to northern communities [59,62]. In 2008, food costs in northern Canada were almost twice as high as those in southern Canadian cities, meaning that even a short-term dependence on retail food in Inuit communities results in large economic consequences for households [59]. Likewise, dietary surveys show that nutrient adequacy suffers on days when country foods are not consumed [61]. Consequently, as described by interviewees and previous studies, even households that are typically food secure may experience transitory food insecurity due to missed subsistence activities attributed to illness [59,65]. It is interesting to note that survey participants who had not recently visited the land or cabin consumed more retail meat and less country meat than those participants who had visited the land or cabin recently; however, they did not spend significantly more money on retail foods. This may indicate that access to country meats allows residents to spend their money on other retail foods, such as fruits and vegetables, whereas residents without access must spend a proportion of their money on meat, thus impacting their nutritional intake [66,67].

Hence, as described by interviewees and other studies, without acknowledging the full value of subsistence activities, investigators have likely underestimated the indirect costs of illnesses in Indigenous communities [20,68]. To acknowledge this issue, subsistence activities and the resultant harvested country foods can be valued by calculating edible weight quantities of country foods and pricing them at the same rate as the nearest equivalent retail food [68–70]. When valued using this substitution cost method, harvests from subsistence activities represent a large component of Arctic community income, emphasizing the need to incorporate such indirect costs into cost-of-illness estimates in these settings [69,70].

As reflected in another study conducted in Rigolet, almost all interviewees reported increasing their consumption of retail foods when sick with AGI to reduce symptom severity [30]. Due to the high retail food costs, this altered consumption could contribute to increased household food expenditures. However, some interviewees reported consuming less food and therefore saving money while sick; therefore, the impact of AGI on food expenditures remains unclear and requires further research. In the broader literature, very few cost-of-illness studies consider such altered household expenditures, demonstrating the need for further case-based studies to capture this information [2].

As reflected in other studies, interviewees reported that subsistence activities are of the utmost importance for Inuit mental, emotional, and spiritual well-being [1,22,25,30]. This concurs with other northern Canadian studies, which also found that decreased paid employment and subsistence activity productivity were linked with poorer physical and mental well-being outcomes [25,27]. As discussed in a previous study conducted in Rigolet, interviewees’ description of AGI severity in terms of lost productivity rather than symptom severity reflects the ‘Role Performance Model’, which suggests that defining health is based on one’s ability to fulfill work and family roles [30,71]. Other studies also reported Inuit individuals describing a sense of pride and cultural connection associated with country food consumption, thus positively impacting mental well-being [31,32,61–63]. However, AGI did not significantly alter survey participants’ overall life satisfaction, suggesting that impacts on mental well-being impacts may be more short- than long-term in nature.

These mental well-being costs are important to consider as decreased mental well-being amplifies the negative impacts of physical illnesses on functionality, thus further impacting quality of life: studies show that the synergistic effect of comorbid decreased mental and physical well-being results in more productivity losses compared to the sum of individual decreases in either mental or physical well-being [16,18]. Moreover, Canadian Inuit have existing high instances of mental health challenges, including higher suicide and addictions levels compared to non-Inuit Canadians; small fluctuations in mental well-being may therefore have more substantial impacts on Inuit as compared to other Canadians [72–74]. Given that the human capital approach typically used to value the indirect costs of illnesses is designed to calculate labour productivity losses, the majority of cost-of-illness studies do not include the intangible costs associated with mental well-being [12,17,55]. Researchers have recently begun using Health Adjusted Life Years (HALYs) to provide a common currency to assess the intangible costs of illnesses and set priorities for resource allocation [75–78]. Disability Adjusted Life Years (DALYs) or Disability Adjusted Life Days (DALDs) are an example of HALYs, in which researchers apply a disease-specific severity weight to the duration of illness, which produces an estimate of the number of healthy years (DALYs) or days (DALDs) lost by an individual due to illness [75]. The DALY and DALD severity weight scales account for functional disability and pain-and-suffering, thus providing a more comprehensive understanding of the indirect costs of illness [75]. Although controversial due to the reliance on subjective valuations, DALYs and DALDs can be translated into a more understandable unit by monetizing them using a specified conversion factor derived from the value of statistical life, which is the value placed on changes in the likelihood of an individual’s death [75,79]. Additional case-based studies are needed to determine the value of DALYs and DALDs within specific settings [77,78].

Interviewees’ reports of the social welfare impact of AGI are reflected by another study conducted in Rigolet, which found that AGI caused sick individuals to miss an average of 0.33 days of usual activities within a 14 day period [24]. Illness can contribute to losses in social welfare and quality of life both directly, as people prefer to be more healthy, and indirectly, by reducing the enjoyment, ability to participate, or utility associated with the consumption of goods and services [80]. Although AGI is a self-limiting illness, the high prevalence of AGI in Rigolet could alter the impacts of AGI on quality of life as compared to elsewhere in Canada [30]. Indeed, previous research in Rigolet highlighted the importance of socializing in the establishment of the highly valued community social networks [30], which is a foundational value underlying Inuit culture [22,81].

Nevertheless, social welfare costs are seldom incorporated into economic evaluations due to the difficulties associated with valuing such activities in monetary terms [5,6,11,15]. Some studies use an opportunity cost approach to value nonmarket activities by using the average wage rate as a proxy for the value of social time lost [5,6,17,55]. Alternatively, future research could investigate the potential of incorporating social welfare losses into the DALY weighting system to value these impacts.

Lastly, interviewees described seasonal variations in the magnitude of specific costs of illness. These impacts were particularly relevant in relation to missing subsistence activities for which the consequences were reportedly more substantial in the spring and summer, as compared to the winter, due to the shorter harvesting season. Participants also described that being sick at particular times during seasons, such as when berries were ripe or when the salmon were running, would have more considerable associated costs. The survey revealed that there were fewer visits to the land or cabin in May compared to September, which could be explained by Inuit using different modes of transportation by season. Additionally, the increased consumption of both country and retail meat in May compared to September may be attributed to September being reserved primarily for berry gathering. Other studies similarly reported seasonal fluctuations in household production, activities, and opportunity costs of time, which translated into variable costs of illnesses [54,82]. Other parameters that influence costs of illness also fluctuate seasonally, including disease incidence, health-seeking behaviours, and country food consumption [63,64,82]. For example, Finner (2015) reported that retail food availability in Rigolet varies seasonally, with less available quantity and variety of fresh foods in the winter and spring months when the community is accessible only by air, which could further influence the seasonality of costs of illness [65]. Additionally, Cunsolo et al. (2013) reported that Rigolet residents’ mental well-being was particularly vulnerable during fall freeze-up and spring thaw, which could therefore exacerbate the mental well-being costs associated with missing subsistence activities during these times [34]. More specifically, the survey results showed that overall life satisfaction was higher in May as compared to September, which could also affect the magnitude of mental well-being costs at certain times of year. Nevertheless, few studies examine the effects of such seasonal fluctuations on the cost of illnesses, thus highlighting the need to carry out cost-of-illness research in all relevant seasons [82].

Our findings suggest that due to their remote location, reliance on the land for physical and mental well-being, and unique culture, Rigolet residents bear many substantial indirect costs of AGI that are not typically experienced in other Canadian non-Indigenous or urban contexts [25,30,61,63,83,84]. Although the magnitude of these community-level indirect costs is small when viewed from a national-scale, these costs greatly influence Inuit food security, mental-wellbeing, social welfare, and overall quality of life, and, in combination with the high burden of AGI [1], perpetuate existing health inequities [18,83,84]. Using community-level perspectives and Western science-based cost-of-illness literature, a case-based context-specific cost-of-illness study framework was designed that is meaningful to both Indigenous and non-Indigenous policy-makers. This comprehensive framework is intended to be used in community-level cost-of-illness studies to generate more robust and accurate estimates of the true costs of AGI in Rigolet. Such estimates could be used by public health decision-makers in order to make evidence-based health policy and resource allocation decisions in order to rectify these inequities [11,20]. For example, findings from this study suggest that transitory food insecurity due to missed subsistence activities is an issue for the Rigolet community. In order to mitigate this concern, the NIHB Plan could include funding or discounts for healthy retail food, or alternatively could coordinate with the community of Rigolet to allow these residents access to the community freezer. However, community perspectives on the most valuable potential changes to the NIHB Plan should be sought prior to implementing any changes.

Qualitative case-based studies are designed to provide an in-depth understanding of individuals’ experiences and perspectives. We gathered extensive data regarding the indirect costs of AGI in Rigolet from 32 interviewees. Nevertheless, this study had some limitations that merit mention. Firstly, this study did not attempt to quantify the indirect costs of AGI, but rather aimed to collect interviewees’ perceptions to identify and characterize these costs. Future studies can incorporate these insights to generate more robust cost-of-illness estimates. Secondly, this study was exploratory; it was conducted in one Inuit community that may not be representative of other Inuit communities meaning that caution should be exercised in generalizing the results. Future research should explore and compare the indirect costs in Rigolet to those in other Inuit communities. Thirdly, longitudinal data were not captured; results may therefore not adequately capture seasonal cost variations. Longitudinal data collection over an entire year would enable more accurate data analysis. Furthermore, the p-values presented in this paper should be considered exploratory in nature due to the small quantitative sample size and power. Nevertheless, the high response rate and no significant differences between demographics between the survey population and the Canadian census suggest a representative sample, which is an important achievement as representative samples in Indigenous populations are often difficult to attain. Fourthly, this study did not assess if costs of illness vary according to gender or age; future research should examine the influence of such demographic factors on costs of illness to inform gender- and age-specific health policies and programs. Finally, AGI cases were self-reported, meaning that recall, interviewer, and misclassification biases are possible.

## Conclusion

This study used a mixed methods design to identify and characterize the indirect costs associated with AGI in Rigolet, Canada. Although there is a well-developed literature on cost-of-illness methods and estimates, few studies examine the costs of illness from a setting-specific perspective or in settings where an acute illness occurs chronically over time in the population, resulting in studies overlooking many important indirect cost contributors. The findings from this study emphasize the importance of considering the numerous indirect cost contributors of AGI in Inuit communities that are perpetuating existing Inuit health inequities, including the economic impacts of missed paid employment, missed subsistence activities, altered diet, decreased mental well-being, decreased social welfare, and seasonality. The results were used to develop a case-based indirect cost-of-illness study framework that accounts for all reported cost contributors, and could be adapted for other Inuit communities and self-limiting illnesses. Using this more comprehensive cost-of-illness study framework, public health decision-makers could obtain a more accurate estimate of the costs of illness within a community, and develop and prioritize funding, policies, and programs accordingly.

This study presents one approach to guide future case-based studies, offering a micro-level understanding of the costs of illness in a specific setting. This approach should be extended to other health research locations to gain a clearer understanding of the various costs of illness in different settings. Although this study offers important information on the indirect costs of illness borne by individuals in Rigolet, future research is needed to quantify these costs and to understand how these costs compare to those in other settings.

## Supporting information

S1 File General interview guides for community members and key informants. (DOCX)

S1 Table Overview of the current cost-of-illness literature with a focus on studies investigating gastrointestinal illnesses or including a variety of indirect cost components. (DOCX)

S2 Table Response rates for quantitative burden of illness survey questions, Rigolet, Canada. (DOCX)

S3 Table Salient quotations from interviewees describing indirect costs of acute gastrointestinal illness in Rigolet, Canada in July 2015. (DOCX)

## References

[1] Harper S, Edge VL, Ford J, Thomas MK, Pearl DL, Shirley J, et al Acute gastrointestinal illness in two Inuit communities: burden of illness in Rigolet and Iqaluit, Canada. Epidemiol Infect. 2015;143: 3048–63. doi: 10.1017/S0950268814003744 25697261 10.1017/S0950268814003744 PMC9151065

[2] Gauci C, Gilles H, O’Brien S, Mamo J, Stabile I, Ruggeri FM, et al Estimating the burden and cost of infectious intestinal disease in the Maltese community. Epidemiol Infect. 2007;135: 1290–1298. doi: 10.1017/S0950268807008084 17313694 10.1017/S0950268807008084 PMC2870703

[3] Sargeant JM, Majowicz SE, Snelgrove J. The burden of acute gastrointestinal illness in Ontario, Canada, 2005–2006. Epidemiol Infect. 2008;136: 451–460. doi: 10.1017/S0950268807008837 17565767 10.1017/S0950268807008837 PMC2870834

[4] Giaquinto C, Van Damme P, Huet F, Gothefors L, Van der Wielen M. Costs of community-acquired pediatric rotavirus gastroenteritis in 7 European countries: The REVEAL Study. J Infect Dis. 2007;195: S36–S44. doi: 10.1086/516716 17539193 10.1086/516716

[5] Henson S, Majowicz S, Masakure O, Sockett P, MacDougall L, Edge VL, et al Estimation of the costs of acute gastrointestinal illness in British Columbia, Canada. Int J Foo. 2008;127: 43–52. 10.1016/j.ijfoodmicro.2008.06.007 18649966

[6] Hellard ME, Sinclair MI, Harris AH, Kirk M, Fairley CK. Cost of community gastroenteritis. J Gastroenterol Hepatol. 2003;18: 322–328. 12603534 10.1046/j.1440-1746.2003.02959.x

[7] Havelaar A, de Wit M, van Koningsveld R, van Kempen E. Health burden in the Netherlands due to infection with thermophilic *Campylobacter* spp. Epidemiol Infect. 2000;125: 505–522. 11218201 10.1017/s0950268800004933 PMC2869634

[8] Thomas MK, Majowicz SE, MacDougall L, Sockett PN, Kovacs SJ, Fyfe M, et al Population distribution of acute gastrointestinal disease in British Columbia, Canada. BMC Public Health. 2006;6: 307 doi: 10.1186/1471-2458-6-307 17178001 10.1186/1471-2458-6-307 PMC1764889

[9] Scharff RL. State estimates for the annual cost of foodborne illness. J Food Prot. 2015;78: 1064–1071. doi: 10.4315/0362-028X.JFP-14-505 26038894 10.4315/0362-028X.JFP-14-505

[10] Hoffmann S, Batz MB, Morris JJG. Annual cost of illness and quality-adjusted life year losses in the United States due to 14 foodborne pathogens. J Food Prot. 2012;75: 1292–1302. doi: 10.4315/0362-028X.JFP-11-417 22980013 10.4315/0362-028X.JFP-11-417

[11] Majowicz S, McNab WB, Sockett P, Henson S, Doré K, Edge V, et al Burden and cost of gastroenteritis in a Canadian community. J Food Prot. 2006;69: 651–659. 16541699 10.4315/0362-028x-69.3.651

[12] McLinden T, Sargeant JM, Thomas MK, Papadopoulos A, Fazil A. Component costs of foodborne illness: a scoping review. BMC Public Health. 2014;14: 509 doi: 10.1186/1471-2458-14-509 24885154 10.1186/1471-2458-14-509 PMC4041898

[13] Zhang W, Bansback N, Anis AH. Measuring and valuing productivity loss due to poor health: A critical review. Soc Sci Med. 2011;72: 185–192. doi: 10.1016/j.socscimed.2010.10.026 21146909 10.1016/j.socscimed.2010.10.026

[14] Creery D, Lyer P, Samson L, Coyle D, Osborne G, MacDonald A. Costs associated with infant bronchiolitis in the Baffin region of Nunavut. Int J Circumpolar Health. 2005;64: 38–45. 15776991 10.3402/ijch.v64i1.17952

[15] Ratcliffe J. The measurement of indirect costs and benefits in health care evaluation: a critical review. Proj Apprais. 1995;10: 13–18.

[16] Kessler RC, Ormel J, Demler O, Stang PE. Comorbid mental disorders account for the role impairment of commonly occurring chronic physical disorders: results from the National Comorbidity Survey. J Occup Environ Med. 2003;45: 1257–1266. doi: 10.1097/01.jom.0000100000.70011.bb 14665811 10.1097/01.jom.0000100000.70011.bb

[17] Mangen M-JJ, Batz MB, Käsbohrer A, Hald T, Morris JG, Taylor M, et al Integrated approaches for the public health prioritization of foodborne and zoonotic pathogens. Risk Anal. 2010;30: 782–797. doi: 10.1111/j.1539-6924.2009.01291.x 19765248 10.1111/j.1539-6924.2009.01291.x

[18] King M, Smith A, Gracey M. Indigenous health part 2: the underlying causes of the health gap. Lancet. Elsevier Ltd; 2009;374: 76–85. doi: 10.1016/S0140-6736(09)60827-8 19577696 10.1016/S0140-6736(09)60827-8

[19] Adelson N. The embodiment of inequity: Health disparities in Aboriginal Canada. Can J Public Heal. 2005;96: S45–S61. 10.1007/BF03403702 PMC6975716 16078555

[20] Usher PJ, Duhaime G, Searles E. The household as an economic unit in Arctic Aboriginal communities, and its measurement by means of a comprehensive survey. Soc Indic Res. 2012;61: 175–202.

[21] Government of Canada. Section 35: Rights of the Aboriginal peoples of Canada. Constitution Act. 1982.

[22] Richmond CAM. The social determinants of Inuit health: a focus on social support in the Canadian Arctic. Int J Circumpolar Health. 2009;68: 471–487. 20044965 10.3402/ijch.v68i5.17383

[23] Inuit Tapiriit Kanatami. Social Determinants of Inuit Health in Canada. Ottawa, Canada; 2014.

[24] Harper SL, Edge VL, Ford J, Thomas MK, Pearl D, Shirley J, et al Healthcare use for acute gastrointestinal illness in two Inuit communities: Rigolet and Iqaluit, Canada. Int J Circumpolar Health. 2015;74: 26290. 10.3402/ijch.v74.26290 PMC4441732 26001982

[25] Cunsolo Willox A, Harper SL, Edge VL, Landman K, Houle K, Ford JD, et al The land enriches the soul: On climatic and environmental change, affect, and emotional health and well-being in Rigolet, Nunatsiavut, Canada. Emot Sp Soc. 2013;6: 14–24.

[26] Petrasek MacDonald J, Cunsolo Willox A, Ford JD, Shiwak I, Wood M, IHACC Research Team, et al Protective factors for mental health and well-being in a changing climate: Perspectives from Inuit youth in Nunatsiavut, Labrador. Soc Sci Med. 2015;141: 133–141. doi: 10.1016/j.socscimed.2015.07.017 26275362 10.1016/j.socscimed.2015.07.017

[27] Harper SL, Edge VL, Ford J, Cunsolo Willox A, Wood M, IHACC Research Team, et al Climate-sensitive health priorities in Nunatsiavut, Canada. BMC Public Health. 2015;15: 605 doi: 10.1186/s12889-015-1874-3 26135309 10.1186/s12889-015-1874-3 PMC4489362

[28] Young TK, Chatwood S. Health care in the North what Canada can learn from its circumpolar neighbours. Can Med Assoc J. 2011;183: 209–214. 21041430 10.1503/cmaj.100948 PMC3033925

[29] Statistics Canada. Rigolet, Newfoundland and Labrador. Census Profile. 2016 Census. Ottawa, Canada; 2016 Feb.

[30] Harper SL, Edge VL, Ford J, Thomas MK, IHACC Research Team, Rigolet Inuit Community Government, et al Lived experience of acute gastrointestinal illness in Rigolet, Nunatsiavut: “Just suffer through it.” Soc Sci Med. 2015;126: 86–98. doi: 10.1016/j.socscimed.2014.12.011 25528558 10.1016/j.socscimed.2014.12.011

[31] Pufall EL, Jones AQ, McEwen SA, Lyall C, Peregrine AS, Edge VL. Perception of the importance of traditional country foods to the physical, mental, and spiritual health of abrador Inuit. Arctic. 2011;64: 242–250.

[32] McGrath-Hanna NK, Greene DM, Tavernier RJ, Bult-Ito A. Diet and mental health in the Arctic: is diet an important risk factor for mental health in circumpolar peoples?—a review. Int J Circumpolar Health. 2003;62: 228–241. 14594198 10.3402/ijch.v62i3.17560

[33] Harper S, Edge VL, Schuster-Wallace CJ, Berke O, McEwen SA. Weather, water quality and infectious gastrointestinal illness in two Inuit communities in Nunatsiavut, Canada: Potential implications for climate change. Ecohealth. 2011;8: 93–108. doi: 10.1007/s10393-011-0690-1 21785890 10.1007/s10393-011-0690-1

[34] Cunsolo Willox A, Harper SL, Ford JD, Edge VL, Landman K, Houle K, et al Climate change and mental health: an exploratory case study from Rigolet, Nunatsiavut, Canada. Clim Change. 2013;121: 255–270.

[35] Cunsolo Willox A, Harper SL, Ford JD, Landman K, Houle K, Edge VL, et al “From this place and of this place:” Climate change, sense of place, and health in Nunatsiavut, Canada. Soc Sci Med. 2012;75: 538–547. doi: 10.1016/j.socscimed.2012.03.043 22595069 10.1016/j.socscimed.2012.03.043

[36] Harper S, Edge VL, Cunsolo Willox A, Rigolet Inuit Community Government. “Changing climate, changing health, changing stories” profile: Using an EcoHealth approach to explore impacts of climate change on inuit health. Ecohealth. 2012;9: 89–101. doi: 10.1007/s10393-012-0762-x 22526749 10.1007/s10393-012-0762-x

[37] Canadian Institutes of Health Research, Natural Sciences and Engineering Research Council of Canada, Social Sciences and Humanities Research Council of Canada. Research Involving the First Nations, Inuit and Métis Peoples of Canada TCPS 2 (2014)—the latest edition of Tri-Council Policy Statement: Ethical Conduct for Research Involving Humans. Ottawa, ON: Government of Canada; 2014 p. 210.

[38] Castleden H, Morgan VS, Lamb C. “I spent the first year drinking tea”: Exploring Canadian university researchers’ perspectives on community-based participatory research involving Indigenous peoples. Can Geogr. 2012;56: 160–179.

[39] Patton MQ. Qualitative Research In: Everitt BS, Howell DC, editors. Encyclopedia of Statistics in Behavioral Science. Chichester, UK: John Wiley & Sons, Ltd; 2005.

[40] Romm NRA. Conducting focus groups in terms of an appreciation of Indigenous ways of knowing: Some examples from South Africa. Forum Qual Soc Res. 2015;16.

[41] Krueger R, Casey M. Focus Groups: A Practical Guide for Applied Research. Los Angeles, California: Sage Publications; 2008.

[42] Sim J. Collecting and analysing qualitative data: issues raised by the focus group. J Adv Nurs. 1998;28: 345–352. 9725732 10.1046/j.1365-2648.1998.00692.x

[43] Duggleby W. What about focus group interaction data? Qual Health Res. 2005;15: 832–840. doi: 10.1177/1049732304273916 15961879 10.1177/1049732304273916

[44] MacDougall C, Baum F. The devil’s advocate: A strategy to avoid groupthink and stimulate discussion in focus groups. Qual Health Res. 1997;7: 532–541.

[45] Seidman I. “Technique isn’t everything, but it is a lot” Interviewing as Qualitative Research: A Guide for Researchers in Education and Social Sciences. 3rd ed. New York: Teachers College Press; 2006.

[46] Kvale S, Brinkmann S. InterViews: Learning the Craft of Qualitative Research Interviewing. Thousand Oaks, CA: SAGE Publications Ltd; 2009.

[47] Braun V, Clarke V. Using thematic analysis in psychology. Qual Res Psychol. 2006;3: 77–101.

[48] Charmaz K. Constructing Grounded Theory: A Practical Guide through Qualitative Analysis. Thousand Oaks, CA: SAGE Publications Ltd; 2006.

[49] DeCuir-Gunby JT, Marshall PL, McCulloch AW. Developing and using a codebook for the analysis of interview data: An example from a professional development research project. Field methods. 2011;23: 136–155.

[50] Harper SL. Gastrointestinal illness in Canada’s North: Implications of climate change on current and future Inuit health University of Guelph 2013.

[51] Majowicz SE, Horrocks J, Bocking K. Demographic determinants of acute gastrointestinal illness in Canada: a population study. BMC Public Health. 2007;7. 10.1186/1471-2458-7-162 PMC1955441 17640371

[52] Creswell JW. Designing and Conducting Mixed Methods Research. Thousand Oaks, CA: Sage Publications; 2007.

[53] Chen Y, Ford L, Hall G, Dobbins T, Kirk M. Healthcare utilization and lost productivity due to infectious gastroenteritis, results from a national cross-sectional survey Australia 2008–2009. Epidemiol Infect. 2015;22: 1–6. 10.1017/S0950268815001375 26095130

[54] Ewing VL, Lalloo DG, Phiri KS, Roca-Feltrer A, Mangham LJ, SanJoaquin MA. Seasonal and geographic differences in treatment-seeking and household cost of febrile illness among children in Malawi. Malar J. 2011;10: 32 doi: 10.1186/1475-2875-10-32 21303538 10.1186/1475-2875-10-32 PMC3049750

[55] Segel JE. Cost-of-Illness Studies—A Primer. 2006;

[56] van den Brandhof W, De Wit G, de Wit M, van Duynhoven Y. Costs of gastroenteritis in The Netherlands. Epidemiol Infect. 2004;132: 211–221. 15061495 10.1017/s0950268803001559 PMC2870096

[57] Oostenbrink J, Koopmanschap M, Rutten F. Guidelines for cost research. Methods and guideline prices for economic evaluations in health care [in Dutch]. Amstelveen; 2000.

[58] Elias D. Models of aboriginal communities in Canada’s north. Int J Soc Econ. 1997;24: 1241–1255.

[59] Ford JD, Beaumier M. Feeding the family during times of stress: experience and determinants of food insecurity in an Inuit community. Geogr J. 2011;177: 44–61. 21560272 10.1111/j.1475-4959.2010.00374.x

[60] Gervais J, Ledrou I. Food insecurity. Heal Reports. 2005;16: 47–51. 15971515

[61] Chan HM, Fediuk K, Hamilton S, Rostas L, Caughey A, Kuhnlein H, et al Food security in Nunavut, Canada: barriers and recommendations. Int J Circumpolar Health. 2006;65: 416–430. 17319086 10.3402/ijch.v65i5.18132

[62] Willows ND. Determinants of healthy eating in Aboriginal peoples in Canada: The current state of knowledge and research gaps. Can J Public Heal. 2005;96: S32–S36. 10.1007/BF03405199 PMC6976035 16042162

[63] Duhaime G, Chabot M, Gaudreault M. Food consumption patterns and socioeconomic factors among the inuit of Nunavik. Ecol Food Nutr. 2002;41: 91–118.

[64] Kuhnlein H V, Soueida R, Receveur OO. Dietary nutrient profiles of Canadian Baffin Island Inuit differ by food source, season, and age. J Am Diet Assoc. 1996;96: 155–162. doi: 10.1016/S0002-8223(96)00045-4 8557942 10.1016/S0002-8223(96)00045-4

[65] Finner KL. Food from here and there, from us and them: Characterizing the food system of Rigolet, Nunatsiavut, Canada. McGill University 2015.

[66] Johnson-Down LM, Egeland GM. How is nutrition transition affecting dietary adequacy in Eeyouch (Cree) adults of Northern Quebec, Canada? Appl Physiol Nutr Metab. 2013;38: 300–305. doi: 10.1139/apnm-2012-0167 23537022 10.1139/apnm-2012-0167

[67] Huet C, Ford JD, Edge VL, Shirley J, King N, IHACC Research Team, et al Food insecurity and food consumption by season in households with children in an Arctic city: A cross-sectional study. BMC Public Health. 2017;17: 1–14. doi: 10.1186/s12889-016-3954-4 28619039 10.1186/s12889-017-4393-6 PMC5472920

[68] Usher PJ. Evaluating country food in the northern Native economy. Arctic. 1976;20: 105–120.

[69] Goldstein JH, Thogmartin WE, Bagstad KJ, Dubovsky JA, Mattsson BJ, Semmens DJ, et al Replacement cost valuation of Northern Pintail (Anas acuta) subsistence harvest in Arctic and sub-Arctic North America. Hum Dimens Wildl. 2014;19: 347–354.

[70] Olar M, Louvel J, Hernandez M, Sauve C, Zussy S, Messier J. Evidence of the socio-economic importance of polar bears for Canada. Ottawa, Canada; 2011.

[71] Elliott-Schmidt R, Strong J. The concept of well-being in a rural setting: understanding health and illness. Aust J Rural Health. 1997;5: 59–63. 9444122 10.1111/j.1440-1584.1997.tb00239.x

[72] Lehti V, Niemelä S, Hoven C, Mandell D, Sourander A. Mental health, substance use and suicidal behaviour among young indigenous people in the Arctic: A systematic review. Soc Sci Med. 2009;69: 1194–1203. doi: 10.1016/j.socscimed.2009.07.045 19700231 10.1016/j.socscimed.2009.07.045

[73] Cunsolo Willox A, Stephenson E, Allen J, Bourque F, Drossos A, Elgaroy S, et al Examining relationships between climate change and mental health in the Circumpolar North. Reg Environ Chang. 2015;15: 169–182.

[74] Cunsolo Willox A, Harper S, Edge V. Examining the climatic and environmental determinants of mental health: A case study from Nunatsiavut, Labrador, Canada. Int J Circumpolar Health. 2013;72: 519–520.

[75] Stein AJ. Rethinking the measurement of undernutrition in a broader health context: Should we look at possible causes or actual effects? Glob Food Sec. 2014;3: 193–199.

[76] Gold MR, Stevenson D, Fryback DG. HALYS and QALYS and DALYS, Oh My: similarities and differences in summary measures of population health. Annu Rev Public Health. 2002;23: 115–34. doi: 10.1146/annurev.publhealth.23.100901.140513 11910057 10.1146/annurev.publhealth.23.100901.140513

[77] Adler M. QALYs and policy evaluation: A new perspective University of Pennsylvania 2005.

[78] FDA. Requirements pertaining to sampling services and private laboratories used in connection with imported food. 2004.

[79] Commission on Macroeconomics and Health. Macroeconomics and health: investing in health for economic development Geneva: Geneva: World Health Organization; 2001.

[80] WHO. WHO guide to identifying the economic consequences of disease and injury. WHO Library Cataloguing-in-Publication Data. Geneva; 2009.

[81] Pauktuutit Inuit Women of Canda. The Inuit Way: A Guide to Inuit Culture. 2006.

[82] Sauerborn R, Nougtara A, Hien M, Diesfeld H. Seasonal variations of household costs of illness in Burkina Faso. Soc Sci Med. 1996;43: 281–290. 8844931 10.1016/0277-9536(95)00374-6

[83] Gracey M, King M. Indigenous health part 1: determinants and disease patterns. Lancet. 2009;374: 65–75. doi: 10.1016/S0140-6736(09)60914-4 19577695 10.1016/S0140-6736(09)60914-4

[84] Stephens C, Nettleton C, Porter J, Willis R, Clark S. Indigenous peoples’ health—why are they behind everyone, everywhere? Lancet. 2005;366: 10–13. doi: 10.1016/S0140-6736(05)66801-8 15993213 10.1016/S0140-6736(05)66801-8

