# Supplementary material for: The hidden costs: Identification of indirect costs associated with acute gastrointestinal illness in an Inuit community
Source: PLoS One. 2018 May 16;13(5):e0196990. doi: 10.1371/journal.pone.0196990 (PMC5955559; doi:10.1371/journal.pone.0196990)
Supplement: S1 File — (DOCX) [file pone.0196990.s001.docx]

**S1 File. General Interview guides for community members and key informants regarding indirect costs of AGI in Rigolet, Canada**

**General Interview Guide: Community Members**

This general interview guide indicates themes to address in interviews with community members regarding the identification and description of the costs associated with acute gastrointestinal illness (AGI) in their community. A separate interview guide provides a similar structure for interviews with key informants (e.g. government officials, health practitioners, public health professionals, community health officials, etc.) regarding their knowledge of the costs of AGI. This guide provides examples of primary questions and follow-up questions that can be posed to the participant(s). This is *not* intended to be a formal survey or interview, but rather a generic guide for leading a conversation about the cost of AGI. The specific questions asked, how they are asked, and the order in which they are asked might differ depending on the participant(s) and context. For example, more in-depth discussion will be prompted for costs that community members identify as key or important, as compared to those costs that participants view as less important.

The purpose of these interviews is to develop a baseline understanding of the nature and quantity of costs that are incurred as a result of AGI in the remote Northern community of Rigolet, Nunatsiavut. The insights from these sessions will complement information from healthcare professionals and the published literature in order to develop a robust framework to assess the economic costs of AGI in Rigolet.

**For each interview, record the following:**

***Number of individuals present:***

***Names of individuals present:***

# Preamble

Thank you for taking the time to attend the interview today. This project aims to establish a study framework to accurately estimate the economic cost of stomach illness in Rigolet. We are conducting interviews with community members to identify the costs that individuals and the community experience when someone is sick with AGI. We also aim to determine how this project can be most useful to the community, and what resources we can develop to best communicate the results. This project is part of IHACC, which is a broader initiative exploring the health adaptations to climate change of Indigenous peoples, which involves a close collaboration between community members, stakeholders, and researchers. An assessment of the economic costs of AGI in Rigolet can be used to identify the most important cost drivers, to facilitate the development of cost-reducing strategies and information sharing about effective cost-control strategies. You can choose not to respond to certain questions and you can withdraw from the study, up until the preliminary data analysis is complete. If you choose to withdraw, your data will not be used.

**All participants are over 18 years of age?** ❑ YES ❑ NO

**Review consent form with all participants** ❑ YES ❑ NO

**Does anyone have any questions before we begin?** ❑ YES ❑ NO

# Part A: Costs of illness

1. When you get sick with a stomach illness, what steps do you usually take?
   1. *Medical professionals: Who do you visit? How do you get there? How far is it? How long does it take? Do you bring anyone? Do you make use of any telehealth initiatives? Who goes/calls? Can you tell me more? Have others had the same or a different experience?*
   2. *Allopathic medications: What medications do you take? How often? For how long? Where do you obtain these medicines?*
   3. *Traditional medications: Can you describe these for me? Where do these come from? Who/how did you learn about these? Are they difficult to obtain? Are there monetary costs associated?*
   4. *Determinants: What factors influence these decisions? Family? Severity? Time of year (seasonality)? Responsibilities? Age? Gender?*

*Activity:* You have each been provided with 5 paper cups and a marker. On these cups, I would like you to think of the five main costs incurred when an individual becomes sick with stomach illness. This may include the costs associated with visiting healthcare professionals, obtaining medicine, accessing traditional medicines, missing hunting or fishing trips, or any other social or economic impact. These costs may be individual or community level costs

*[Skip the following step if it is an individual interview]* Now that everyone has five cups each labelled with a cost, I would like the group to try and consolidate these costs into 6 to 8 categories. You can stack the cups that are being collapsed into a single category, and the categories can be renamed as a group if desired.

*[If it is an individual interview, provide 25 jellybeans to the individual]* Now that we have these main categories, among the group you will now be given 100 jellybeans. I would like you as a group to divide these jellybeans among the cups, with each jellybean representing the specific importance attributed to that particular cost. I.E. More jellybeans means that this particular cost is more important. As a group you will have to decide what “importance” means. This may be the biggest cost, the biggest emotional cost, etc.

During this activity, ensure that you understand why specific costs are being ranked in the order they are (why did they rank them in this way?). If necessary, the following prompts will be used:

1. When you get sick with a stomach illness, what kinds of traditional activities might you miss? What does this mean for you and your family?
   1. *Hunting? Trapping?*
   2. *Fishing?*
   3. *Community activities?*
   4. *Social impacts? (mental health, changed social relationships, etc.)*
   5. *What is the cost associated with each of these?*
2. When you get sick with a stomach illness, do you eat additional/more expensive/different foods?
   1. *Country versus store bought foods*
   2. *Cost associated with each of these?*
3. Are there long-term impacts associated with becoming sick with AGI?
   1. *Economic?*
   2. *Social?*
   3. *Lifestyle changes?*

# Part B: Application of results

1. Is it useful for you to have the information gained from this research? If so, how would you like to learn about this information? What resources do you think should be developed to make the results of this study accessible to community members?
   1. Video?
   2. Pamphlet?
   3. Community input tool
   4. Other
2. Who else do you think should be made aware of this information?

# Wrap-up

These are all of my questions, but do you have any comments to add or were there questions you thought I would ask but didn’t? Are there any important points or topics that didn’t come up?

*Summarize the discussion and ask*: Is this an accurate summary?

Thanks again for your time. If you have any questions or concerns regarding today’s interview, please do not hesitate to contact me, any member of the research team, or the University of Guelph’s research ethics office.

| **General Prompts: Each time a specific cost category or stakeholder is mentioned:**   \| ***Costs*** \| - Who bears the cost? \| - How many units? \| - Over what length of time? \| - Data source for unit cost? \| \| --- \| --- \| --- \| --- \| --- \| \| ***People*** \| - What is their interest? \| - Contact info? \| |
| --- | --- | --- | --- | --- | --- | --- | --- | --- |

**General Interview Guide: Health Professionals**

This general interview guide indicates themes to address in interviews with key informants (e.g. government officials, health practitioners, public health professionals, community health officials etc.) regarding the identification and quantification of the costs of acute gastrointestinal illness (AGI) in Rigolet, Nunatsiavut. A separate interview guide provides a similar structure for interviewing community members about their experiences with the costs of AGI. Examples of primary interview questions and follow-up questions are provided in this guide. This is *not* intended to be a formal survey, but rather a generic guide for a qualitative conversation about the cost of AGI. The specific questions asked, how they are asked, and the order in which they are asked will differ in each interview according to the interview subject and context. For example, questions in interviews with officials working in a specific aspect of healthcare (e.g. public health, medical administration, medical practice etc.) will be specific to that individual’s knowledge of the healthcare costs of illness. The level of detail will also differ according to the interview subject. For example, someone involved at the community level would be asked specific questions about community-level costs, whereas a government official working in a regional centre would be asked broader questions about aggregate economic implications.

The purpose of this interview guide is to develop a baseline understanding of the nature and quantity of economic costs that are incurred as a result of AGI in the remote Northern community of Rigolet, Nunatsiavut. These interviews will complement the information from community members and the published literature in order to develop a robust framework to assess the economic costs of AGI in Rigolet.

**For each interview, record the following:**

***Organization the individual represents:*** national, regional, local / municipal government; health practitioner (i.e. doctor, nurse etc); NGO; traditional health provider, researcher

***Individual’s role in the organization:***

***Number of years in the position:***

# Preamble

Thank you for taking the time to meet with me today. This project aims to establish a study framework to accurately estimate the economic cost of AGI in Rigolet. We are interviewing public health and medical professionals to identify and quantify economic costs of illness, and to determine the ways in which this project can be most useful to practitioners and policymakers. This project is part of a broader initiative exploring the health adaptations to climate change of Indigenous peoples, which involves a close collaboration between community members, stakeholders, and researchers. An assessment of the economic costs of AGI in Rigolet can be used to identify the most important cost drivers, to facilitate the development of cost-reducing strategies and information sharing about effective cost-control strategies. If at any point you don’t want to answer a question, please feel free to skip it. If you say something that you don’t want recorded, just say so, and it can be removed, even after the interview is complete. You can withdraw from the research project at any time, and you can withdraw your interview, up until the preliminary data analysis is complete and you have been offered an opportunity to review any quotes we use from your interview in the context of our research findings.

**Participant is over 18 years of age?** ❑ YES ❑ NO

**Review consent form with participant** ❑ YES

**Does anyone have any questions before we begin?** ❑ YES ❑ NO

# building rapport: participant introductions

1. Can you tell me about yourself, describing your areas of expertise and interest?
2. Can you describe any general issues of Indigenous health that you have explored professionally, or that interest you?

One of the major objectives of this project is to establish the costs of AGI. So I have some questions for you about what costs might be incurred, by individuals and organizations, both direct and indirect, when an individual becomes sick with AGI.

# Part A: Cost identification

1. What are some of the costs that an individual might incur when they become ill with AGI?
   1. *Medicine (allopathic or traditional)?*
   2. *Visits to a professional?*
   3. *Transportation?*
   4. *Missed activities?*
   5. *Social impacts? (Mental health, changed social relationships, etc.)*
2. How often does someone from the community travel South for medical treatment for stomach illness? What does this entail?
   1. *Accompaniment?*
   2. *Medevac versus regular flight*
   3. *Translation services needed?*
   4. *Other expenses? (Accommodation or other costs for accompaniment?)*
   5. *Children at home?*
   6. *Is there a community response?*
3. Are there any costs that your organization/unit incurs when an individual in a remote community contracts AGI? If so, can you describe these costs?
   1. *Who pays for what?*
4. Are you aware of any data sources or resources regarding the costs that we have identified?
   1. *Documents, reports, websites, or other data sources?*
   2. *How to access?*

Once we have completed our data collection and analysis, we would like to ensure that these results are made available to professionals and the public to be used to inform policies and programs. As such, my last few questions relate to the application of our results.

# Part B: application of results

1. How do you think the results of this project could be useful to you in your position?
   1. *Risk prioritization*
   2. *Cost drivers*
   3. *Cross-community comparison*
2. In order to communicate our results, are there specific resources that would be most effective?
   1. *Detailed report, video, pamphlet, published paper*

# Wrap-up

1. Is there anything else that you thought of during our conversation that you want to add?
   1. *Documents, website, or other sources of information?*
   2. *Trends or costs that could become more important in the future?*
2. Are there any other individuals, organizations, or stakeholders that should be contacted as part of this project?

This concludes my questions, but do you have any comments to add or were there questions you thought I would ask but didn’t? Are there any important points or topics that didn’t come up?

Thanks again for your time. If you have any questions or concerns regarding today’s interview please do not hesitate to contact me, any member of the research team, or the University of Guelph’s research ethics office.

| **General Prompts: Each time a specific cost category or stakeholder is mentioned:**   \| ***Costs*** \| - Who bears the cost? \| - How many units? \| - Over what length of time? \| - Data source for unit cost? \| \| --- \| --- \| --- \| --- \| --- \| \| ***People*** \| - What is their interest? \| - Contact info? \| |
| --- | --- | --- | --- | --- | --- | --- | --- | --- |
