# Supplementary material for: The hidden costs: Identification of indirect costs associated with acute gastrointestinal illness in an Inuit community
Source: PLoS One. 2018 May 16;13(5):e0196990. doi: 10.1371/journal.pone.0196990 (PMC5955559; doi:10.1371/journal.pone.0196990)
Supplement: S1 Table — (DOCX) [file pone.0196990.s002.docx]

**S1 Table. Overview of the current cost-of-illness literature with a focus on studies investigating gastrointestinal illnesses or including a variety of indirect cost components**

| **Study** | **Research setting** | **Illness under study** | **Inclusion and quantification of indirect costs** |
| --- | --- | --- | --- |
| **Cost-of-illness studies investigating gastrointestinal illness** | | | |
| Chen et al., 2015 | Australia | Infectious gastroenteritis | Lost productivity—did not quantify |
| Friesema et al., 2012 | Netherlands | Gastroenteritis | Lost productivity—assigned average wage rate value for adults |
| Gauci et al., 2007 | Malta | Infectious intestinal disease | Lost productivity—divided the population into six cohorts using education level, then assigned average wage rate for adults within each of these cohorts  Telephone calls, special foods and rink, leisure items, new clothing, new bedding, cleaning materials—assigned market value to each item |
| Giaquinto et al., 2007 | Belgium, France, Germany, Italy, Spain, Sweden, and the United Kingdom | Pediatric rotavirus gastroenteritis | Lost productivity—assigned average wage rate for adults providing caregiving services or average daily absenteeism allowance  Extra diapers—assigned market values |
| Hellard et al., 2003 | Australia | Gastroenteritis | Lost productivity—assigned average wage rate for adults  Lost non-paid employment—assigned average working wage rate for adults |
| Henson et al., 2008 | British Columbia, Canada | Acute gastrointestinal illness | Lost productivity—assigned average wage rate for adults |
| Hoffmann et al., 2012 | United States of America | Foodborne illnesses | Lost productivity—assigned age-adjusted daily wage rate using the average daily wage rate and the employment factor  Premature mortality—estimated mean QALY losses, however did not incorporate into the cost of illness estimate |
| Majowicz et al., 2006 | Hamilton, Ontario | Gastroenteritis | Lost productivity—assigned average wage rate for adults |
| Scharff, 2015 | United States of America | Foodborne illnesses | Lost productivity—created a model that accounts for compensation costs, labor force participation, and expected lost work days associated with specific illnesses  Lost utility (well-being)—monetized losses in Quality-Adjusted Life Years using state-specific estimates for the Value of Statistical Life |
| Van den Brandhof et al., 2004 | Netherlands | Gastroenteritis | Lost productivity—divided the population into age and gender cohorts then assigned average wage rate for each cohort |
| **Cost-of-illness studies including a variety of indirect cost components** | | | |
| Creery et al., 2004 | Baffin region of Nunavut, Canada | Bronchiolitis and viral pneumonia | None |
| Ewing et al., 2011 | Chikhwawa district of Malawi | Pediatric febrile illness | Seasonal impacts—collected data in two seasons  Lost productivity—assigned minimum wage rate value for adults with no professional training and living in a rural area |
| Sauerborn et al., 1996 | Kossi Province, Burkina Faso | All illnesses | Seasonal impacts—collected data in two seasons (at six times)  Lost productivity—divided the population into age cohorts, then assigned average shadow-wage rate for each age cohort |
