# Supplementary material for: The hidden costs: Identification of indirect costs associated with acute gastrointestinal illness in an Inuit community
Source: PLoS One. 2018 May 16;13(5):e0196990. doi: 10.1371/journal.pone.0196990 (PMC5955559; doi:10.1371/journal.pone.0196990)
Supplement: S2 Table — (DOCX) [file pone.0196990.s003.docx]

**S2 Table.** Response rates for quantitative burden of illness survey questions, Rigolet, Canada

| **Variable** | **Response rate** |
| --- | --- |
| Missed subsistence activity | 100% |
| Recent visit to the cabin | 100% |
| Overall life satisfaction | 100% |
| AGI case status | 99.6% |
| Number of meals including country food | 97.8% |
| Money spent on retail food | 96.5% |
| Number of meals including retail food | 94.8% |
| Money spent on country food | 88.5% |
