# Supplementary material for: The hidden costs: Identification of indirect costs associated with acute gastrointestinal illness in an Inuit community
Source: PLoS One. 2018 May 16;13(5):e0196990. doi: 10.1371/journal.pone.0196990 (PMC5955559; doi:10.1371/journal.pone.0196990)
Supplement: S3 Table — (DOCX) [file pone.0196990.s004.docx]

**S3 Table. Salient quotations from interviewees describing indirect costs of acute gastrointestinal illness in Rigolet, Canada in July 2015**

| Description of acute gastrointestinal illness (AGI) indirect costs: exemplar supporting interview quotations | |
| --- | --- |
| **Factors influencing indirect economic costs** | |
| Missed paid employment | - “Well you wouldn’t be working, so you wouldn’t be putting anything into the economy or getting anything out of the economy, so yeah I think there would be an impact on the economy, but not as much as your mental wellness.” - “If it’s bad enough, I mean you miss out on everything. Work or whatever you want to do.” - “The economic issue is big.” |
| Caregiving | - “Some of us got spouses that work in like Goose Bay or something and are gone for two weeks, so you got no choice sometimes if your spouse is gone, you’ve got to stay home.” |
| Sick leave coverage | - “Well if it’s a job you don’t have leave for, I guess you’ll lose income” - “Well if you’re, say you are a casual worker and you have to miss work, they aren’t going to pay you for those days.” - “Well not so much the [economic impact of the] work part, you have sick days for that.” |
| Missed subsistence activities | - “And I can see it preventing people from going hunting or going to the cabin as well. Who wants to go to the cabin when they’re sick? Cause you’ll never know if it’s going to get worse or if it’s just going to pass. So I wouldn’t go to the cabin if I were sick.” - “I think people do sometimes too though miss a hunting trip or something because they have bad stomach or are sick.” - “Going out on the land, going to the cabin, you wouldn’t want to be down there when you’re stomach sick, you know?” |
| Seasonality | - “[Missing subsistence activities] can actually be quite impactful to what it is that they do. Because if you know you have a certain window for something like goose hunting in October and you miss that window because you are at home treating upset stomach and different symptoms that you’re experiencing because of an AGI illness, that’s a very big subsistence kind of activity that you’ve missed out on. And it lasts for a very short period of time, it’s a very small window. So it depends on the activity of course. When it comes to fishing for salmon and char and things like that, that also impacts not only that period of time but throughout the winter as well because that’s the winter stores of food for a lot of families, it is how they get their proteins. And berries and things like that that actually sustain them through the winter months. The store is there but selection is limited and so those kinds of activities are very important.” - “If you were sick for a while, just if you’re sick for two months of the years, like June and July, then you only have a little time left before the snow comes again or it starts freezing." - “Say if the fish are running, say for example, or the berries are ripe today or tomorrow, and if you’re not feeling well enough to go, you might not get the opportunity when you’re better to go, right?” - “And it depends on what time of year it is, if it’s berry picking time and you’re the one picking berries then you’d have to have someone else do that, or you just wouldn’t have any.” |
| Food security | - “[If I’m sick] I won’t get my char. I won’t get my trout. I’ll get some of it, but I won’t get it all. I usually do at least 2 trips to get my char, probably another trip to get bake apples, and then at least 1 trip late in August to get char again and trout with my cousin. If I get sick and lose either one of those periods then I’ll either not get my berries, or not get my char, or some trout” - “My husband has some issues with his stomach, but it’s a condition, and he didn’t get to pick berries. Well then I had to buy them from someone to make up the slack.” - “If you can’t [get the subsistence harvest] then you will either go without or buy from others” |
| Food costs | - “But then if it’s one of those bad years and you have to scrape for the bake apples, generally go to another community and buy them, but we tend not to do that because, single dad so you’re money only stretches so far, so you only do what you can.” - “Yeah, sometimes [individuals’ diets when sick with AGI] can be more expensive, yeah. It depends on what you have to buy, right? And especially buying it here, because it’s very very expensive here.” |
| Altered diet | - “Like if they had a stomach illness they would probably just eat bananas, like applesauce, whatever something that’s not too heavy.” - “When I myself have the stomach bug or whatever, I just don’t eat at all, I just drink plenty of fluids and try and rehydrate myself and not cause any upset stomach issues or anything. So I don’t eat, I just drink and rest.” - “I just don’t eat as much when I’m sick.” |
| **Mental well-being costs** | |
| Mental well-being impacts | - “Well in addition to say someone missed out and they could have come back with 15 ducks and 7 geese, that would have fed their family for months, not only that but there’s the mental health component. A lot of people feel that restores a sense of balance when they go out on the land, and they feel connected.” - “Maybe its more of a conscious thing, if it happens quite frequently there is a social impact, people begin to worry, and you know I guess, become stressed about it.” |
| **Social welfare costs** | |
| Missed social or cultural experiences | - “You can’t be around others when you’re sick, but I mean there are a lot of social things that you can miss that are really important for the family, like holidays, Christmas events, things like that, or graduations, or baptisms, or whatever people feel are important to them.” - “And then if you’re taking your kids out for that whole experience, then you’re missing that too if you can’t go.” - “You know if you’re not feeling well, you’re not sleeping well, or eating well, and all these things sort of come around to generally missing a lot of social activities and family time.” - “It can cause you to miss important things […] like say if we have a women’s program and we are going off on the land, someone gets a stomach illness then they’ll stay home, they’ll miss out.” |
